# Calcified Appendiceal Phytobezoar Associated With Complicated Acute Appendicitis: A Case Report

**DOI:** 10.7759/cureus.117630

**Published:** 2026-10-08

**Authors:** Sergio Miguel Suárez Fuentes, María N Sandoval Arreola, Mauro A Cardenas, Soledad Merced Castro Olmedo, José J Cervantes De Anda, Claudia C Gómez Manzo, Jael Yamelis Gutierrez Luna, Daniel H Rodríguez Cárdenas, Diana Arias Galaviz, Juan de la Cruz Ramírez Jaimez

**Affiliations:** 1 General Surgery, Hospital Regional Valentín Gómez Farías, Instituto de Seguridad y Servicios Sociales de los Trabajadores del Estado (ISSSTE), Zapopan, MEX; 2 Surgery, Hospital General Presidente Lazaro Cardenas del Rio, Instituto de Seguridad y Servicios Sociales de los Trabajadores del Estado (ISSSTE), Chihuahua, MEX; 3 Pathology, Hospital Regional Valentín Gómez Farías, Instituto de Seguridad y Servicios Sociales de los Trabajadores del Estado (ISSSTE), Zapopan, MEX

**Keywords:** acute appendicitis, appendiceal phytobezoar, appendicolith, calcified bezoar, histopathology, laparoscopic appendectomy, periappendiceal abscess

## Abstract

An appendiceal phytobezoar is an uncommon intraluminal aggregate of indigestible plant material that may resemble a conventional appendicolith when calcified. We describe a 34-year-old woman who presented after approximately one day of severe right lower quadrant abdominal pain accompanied by nausea and vomiting. She was afebrile and hemodynamically stable but had localized peritoneal signs and neutrophilic leukocytosis. Plain radiographs showed a right lower quadrant radiopacity, and noncontrast computed tomography demonstrated a large appendiceal calcification with surrounding inflammatory changes. Emergency laparoscopy revealed a markedly enlarged, inflamed appendix and a localized purulent collection adjacent to an anatomically intact appendiceal base. Adhesiolysis and drainage of the collection were followed by laparoscopic appendectomy, securing the stump with two endoloops. The specimen measured 7.0 cm in length and 3.5 cm in maximum diameter and contained a 1.7 × 1.2 cm intraluminal concretion. Histopathology established suppurative and necrotizing acute appendicitis with ulceration and identified the concretion as a phytobezoar composed of vegetable fibers and dystrophic calcification. A Blake drain was removed on postoperative day 7 after drain output had ceased; subsequent follow-up remained favorable. This case illustrates the diagnostic limitation of labeling every calcified appendiceal concretion a fecalith and the value of radiologic-operative-pathologic correlation in symptomatic patients with apparently rapid progression to complicated appendicitis.

## Introduction

Acute appendicitis is a frequent abdominal surgical emergency, but its underlying causes and morphologic severity are heterogeneous. Clinical evaluation supported by imaging guides risk stratification, and laparoscopic appendectomy remains a standard operative approach when surgery is indicated. The 2025 World Society of Emergency Surgery (WSES) Jerusalem guideline update also emphasizes distinguishing uncomplicated disease from abscess, gangrene, or perforation because the distinction affects treatment and postoperative management [1].

Luminal concretions are frequently described as appendicoliths or fecaliths. In contrast, a phytobezoar is an aggregate of poorly digested vegetable material; when confined to the appendix, the term microphytobezoar has been used even for small intraluminal collections. Engin et al. identified undigested plant residue in seven appendectomy specimens (0.35% of the reported cohort), of which two had appendiceal inflammation; the remaining cases had obstruction and lymphoid hyperplasia. Such observations establish that plant-derived material may lodge in the appendix, although its presence does not establish that it initiated every episode of appendicitis [2]. A systematic review of adult appendiceal foreign-body case reports further demonstrates the diversity of uncommon intraluminal materials encountered in appendiceal disease [3].

Other bezoar types, including trichobezoars, have been described in association with acute appendicitis [4]. Conversely, calcified appendiceal concretions are compositionally diverse, and radiologic density alone cannot determine whether a stone is primarily fecal, vegetable, or mixed [5]. We report a histopathologically identified, dystrophically calcified appendiceal phytobezoar in a patient with suppurative-necrotizing appendicitis and an operatively identified periappendiceal purulent collection, supported by sequential radiographic, tomographic, laparoscopic, gross, and microscopic documentation.

## Case presentation

Clinical presentation

A 34-year-old woman without known chronic disease presented to the emergency department in September 2026 after developing severe, sharp right iliac fossa pain approximately one day earlier. She rated the pain intensity as 10/10 on the visual analog scale (VAS) and reported associated nausea and vomiting but denied fever. Her history included an elective cesarean delivery 11 years previously. She denied routine medications, medication allergies, tobacco use, alcohol consumption, and illicit drug use. Her mother had type 2 diabetes mellitus.

On surgical assessment, she was alert, cooperative, and hemodynamically stable. Blood pressure was 137/91 mmHg, heart rate 70 beats/min, respiratory rate 20 breaths/min, temperature 36.8°C, and oxygen saturation 98%. Physical examination revealed a soft abdomen with marked superficial and deep tenderness in the right lower quadrant, positive McBurney point tenderness, and rebound tenderness (positive Blumberg sign), consistent with localized peritoneal irritation. Rovsing, psoas, and Dunphy signs were also reported as positive in the admission assessment. Admission testing demonstrated leukocytosis with neutrophilia; the complete recorded laboratory panel is presented in Table 1.

**Table 1:** Admission laboratory results. Reference ranges correspond to standard adult international reference intervals. ALT, alanine aminotransferase; aPTT, activated partial thromboplastin time; AST, aspartate aminotransferase; BUN, blood urea nitrogen; INR, international normalized ratio; LDH, lactate dehydrogenase

Parameters	Patient values	Reference range (with units)
Hemoglobin	11.4	12.0-16.0 g/dL
Hematocrit	34.9	36-48%
Platelets	402	150-400 ×10³/µL
Leukocytes	17.9	4.5-11.0 ×10³/µL
Absolute neutrophils	14.24	1.5-7.5 ×10³/µL
Prothrombin time	13.1	11.0-13.5 s
aPTT	27.7	25-35 s
INR	1.131	0.8-1.2
Glucose	106	70-100 mg/dL
BUN	6.54	6-20 mg/dL
Urea	14	13-43 mg/dL
Creatinine	0.49	0.5-1.1 mg/dL
Calcium	10.0	8.5-10.2 mg/dL
Phosphorus	3.8	2.8-4.5 mg/dL
Chloride	101.7	96-106 mmol/L
Potassium	3.8	3.7-5.2 mmol/L
Sodium	136	135-145 mmol/L
Magnesium	1.85	1.7-2.2 mg/dL
ALT	26	4-36 U/L
AST	26	8-33 U/L
Alkaline phosphatase	69	20-130 U/L
Total bilirubin	0.62	0.1-1.2 mg/dL
Direct bilirubin	0.15	0.0-0.3 mg/dL
Indirect bilirubin	0.47	0.2-0.9 mg/dL
Total protein	8.75	6.0-8.3 g/dL
Albumin	5.41	3.4-5.4 g/dL
Globulin	3.34	2.0-3.5 g/dL
LDH	224	105-333 U/L

Diagnostic assessment

Frontal and lateral plain pelvic/abdominal radiographs demonstrated a conspicuous calcified focus projecting over the right lower quadrant. Electronic measurements on the available frontal and lateral images were approximately 10.31 × 14.58 mm and 10.25 × 17.59 mm, respectively; these are projection-dependent measurements rather than definitive specimen dimensions (Figure 1).

**Figure 1:**
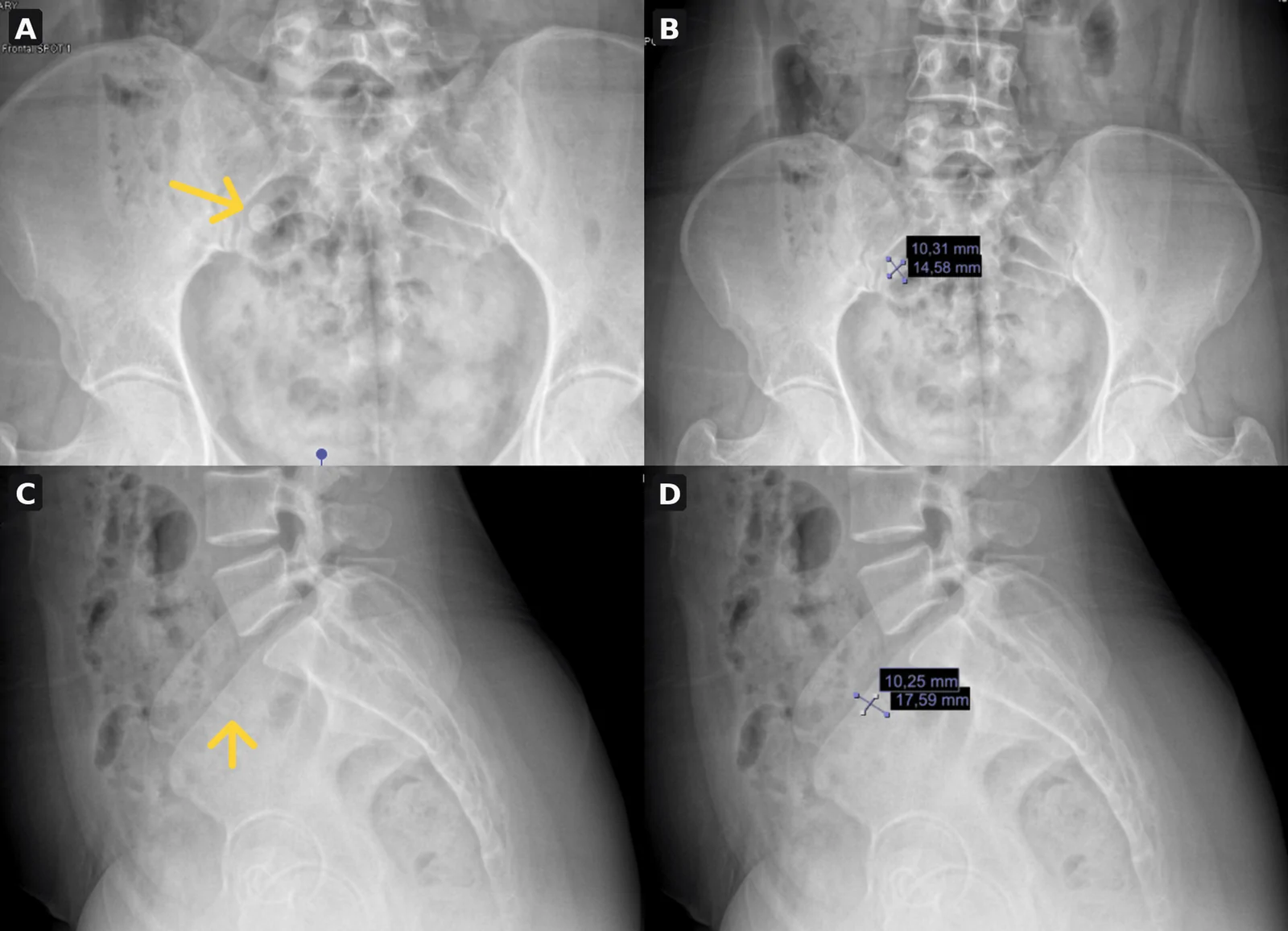
Preoperative frontal and lateral radiographs demonstrating an appendiceal-region calcification. (A) Frontal radiograph showing a conspicuous calcified focus projected over the right lower abdominal quadrant. The yellow arrow points to the right lower quadrant calcification, subsequently identified as the calcified appendiceal phytobezoar. (B) Frontal radiograph with the same calcific focus electronically measured in projection (approximately 10.31 × 14.58 mm). (C) Lateral radiograph showing the corresponding calcified focus. The yellow arrow identifies the same calcified focus. (D) Lateral radiograph with the same calcific focus electronically measured in projection (approximately 10.25 × 17.59 mm). The electronic measurements are projection dependent and do not represent the definitive specimen dimensions.

Noncontrast abdominopelvic computed tomography showed a dense appendiceal concretion, periappendiceal fat stranding, and inflammatory enlargement of the appendix; the admission description also noted periappendiceal fluid. The annotated axial image measured the concretion at approximately 13.07 × 10.36 mm in that plane, whereas the treating team's three-dimensional estimate in the admission note was approximately 1.0 × 1.3 × 1.7 cm. No formal radiologist's interpretation was available in the material supplied for this report. The clinical and imaging findings supported acute appendicitis, and urgent laparoscopic appendectomy was selected (Figure 2).

**Figure 2:**
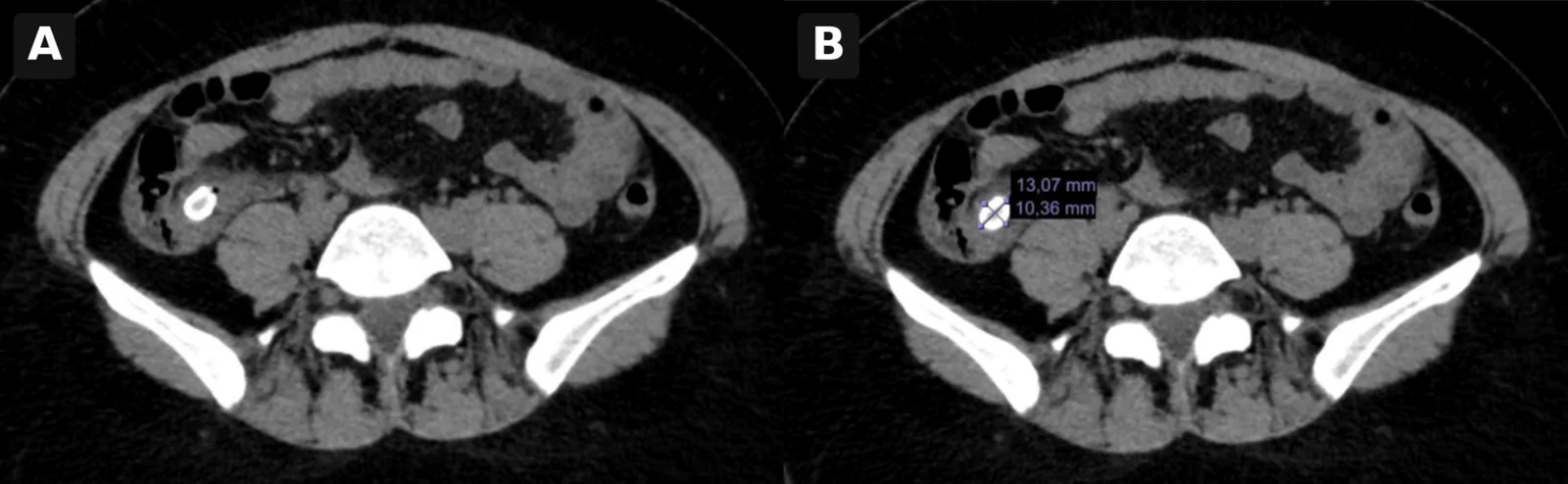
Noncontrast computed tomography demonstrating the appendiceal concretion. (A) Axial noncontrast abdominopelvic computed tomography showing a conspicuous hyperdense concretion in the right lower quadrant at the inflamed appendix. (B) Annotated axial image measuring approximately 13.07 × 10.36 mm at this level.

Operative findings and treatment

Under general anesthesia, pneumoperitoneum was established through an umbilical Veress needle, followed by umbilical camera access and working ports in the suprapubic area and left iliac fossa. Laparoscopic exploration revealed a markedly enlarged and inflamed vermiform appendix surrounded by dense inflammatory tissue and adhesions. A localized purulent collection, considered macroscopically consistent with a periappendiceal abscess, was present adjacent to the appendiceal base. During careful adhesiolysis, the collection opened and purulent material spilled locally; it was promptly aspirated and the affected region cleaned. Further dissection exposed an anatomically intact appendiceal base and separated the mesoappendix. The appendicular artery and mesoappendix were divided with an ultrasonic energy device. Two endoloops were placed to secure the appendiceal stump before the appendix was divided, with no macroscopic stump leakage or active bleeding on final inspection. A Blake drain was left directed toward the cecal region and adjacent paracolic gutter. The specimen was extracted and submitted for histopathology (Figure 3).

**Figure 3:**
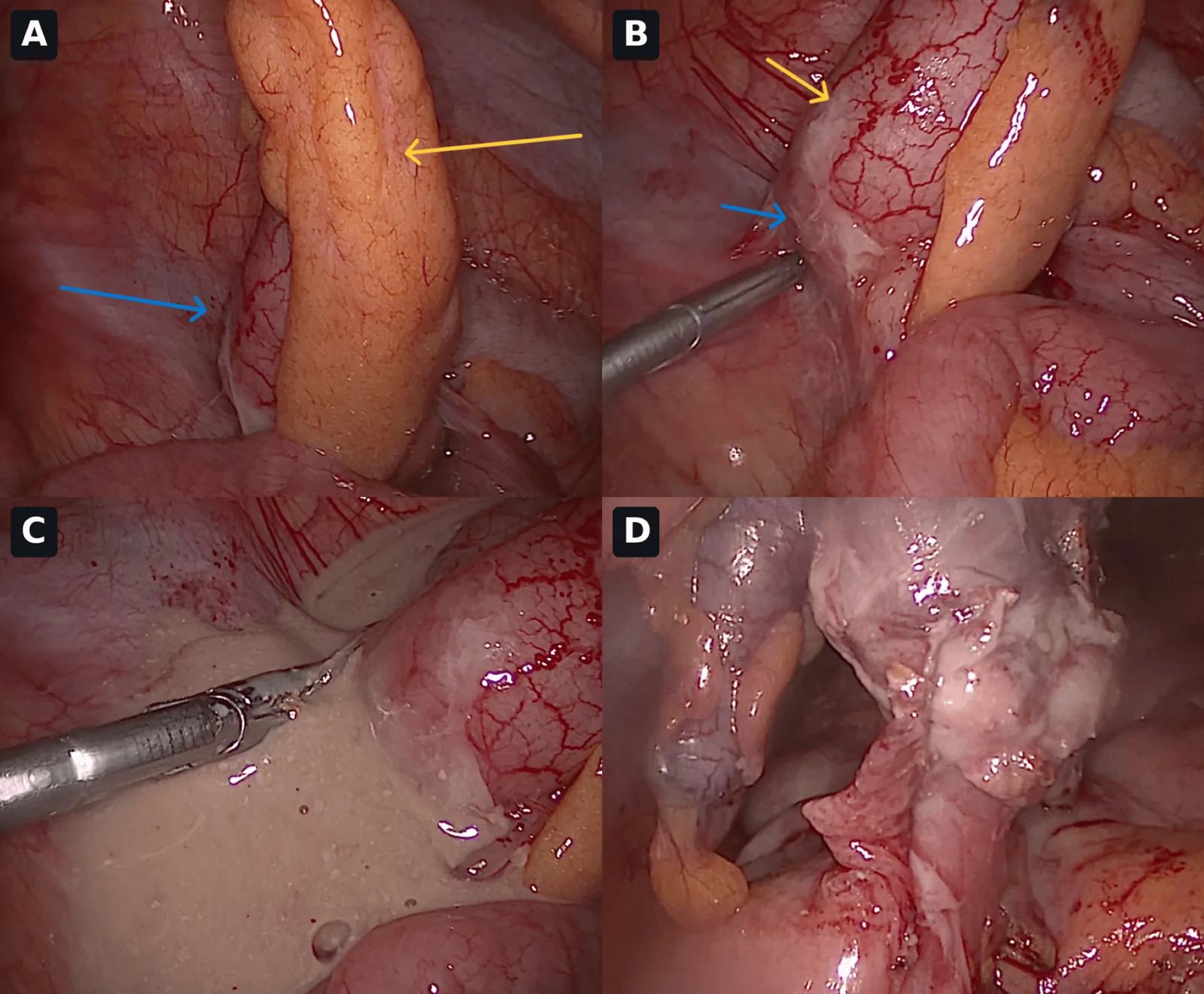
Operative laparoscopic sequence of complicated acute appendicitis. (A) Markedly enlarged and inflamed appendix (blue arrow) with its adjacent mesoappendix (yellow arrow). (B) Inflamed appendiceal base, with the blue arrow identifying the location of the localized purulent collection; the yellow arrow denotes a suspected, not directly visible, position of the intraluminal concretion. (C) Purulent material visible after the localized collection was opened during dissection; the material was subsequently aspirated. (D) Appendix and base exposed after careful dissection of adhesions and mesoappendix, with an enlarged area corresponding to the suspected concretion site. The images do not by themselves establish spontaneous perforation.

Macroscopic and microscopic pathology

Gross examination showed an appendix measuring 7.0 cm in length and 3.5 cm in maximum diameter, with a yellow-brown congested external surface. On opening, a 1.7 × 1.2 cm lobulated, yellow intraluminal concretion was identified. A fibrotic, ulcerated, stenotic area was described near the appendiceal orifice. Specimen photographs documented the concretion in situ and the corresponding intraluminal space after removal, as well as the intact concretion and its cut surface against a metric scale (Figure 4).

**Figure 4:**
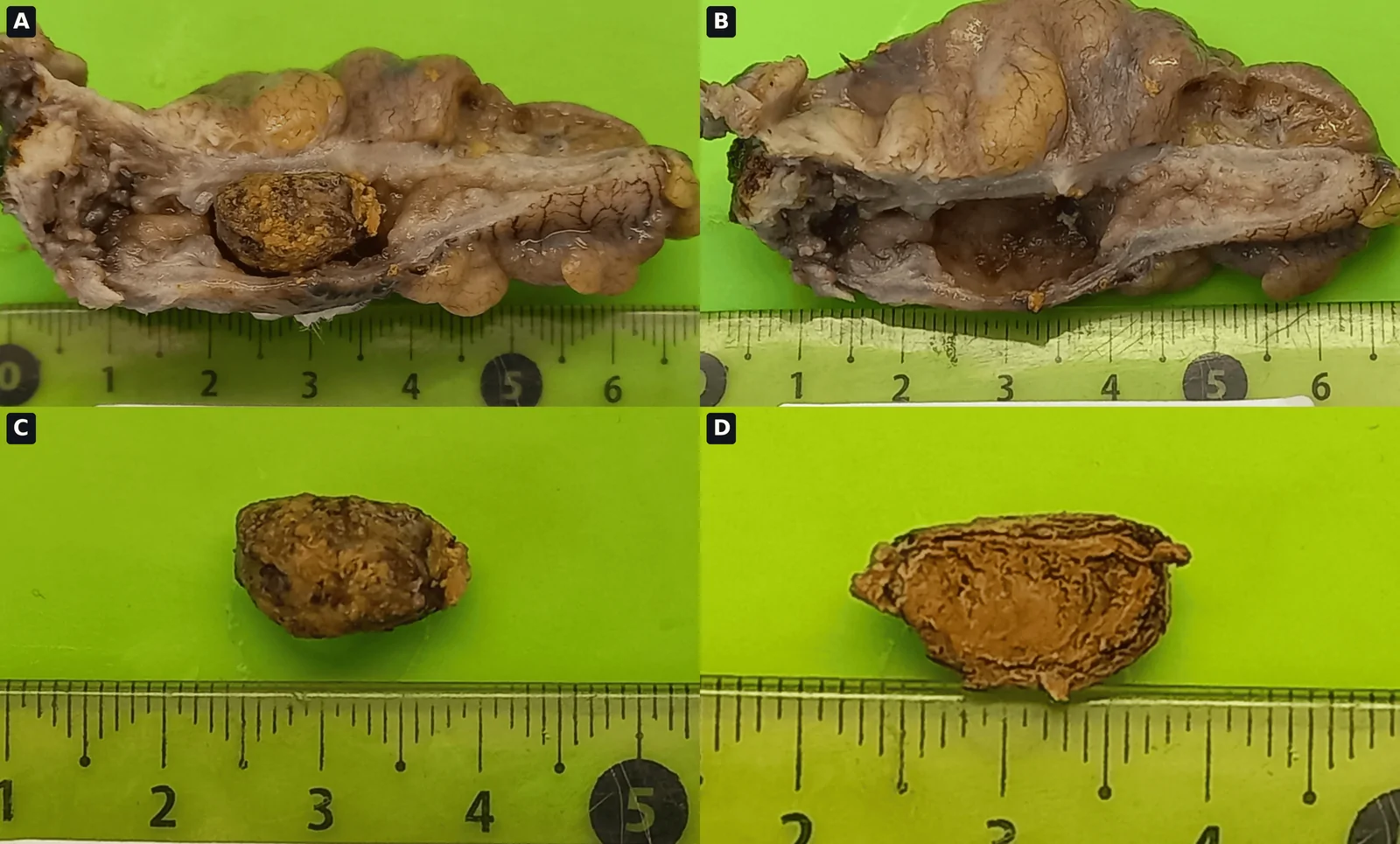
Gross correlation of the appendectomy specimen with the calcified phytobezoar. (A) Longitudinally opened appendectomy specimen containing the intraluminal concretion in situ, with a metric scale. (B) The same specimen after removal of the concretion, revealing the corresponding intraluminal space. (C) Intact extracted phytobezoar beside a metric scale. (D) Longitudinally sectioned phytobezoar demonstrating its internal appearance; the official gross measurement was 1.7 × 1.2 cm.

The histopathology report diagnosed acute suppurative and necrotizing appendicitis with follicular features, ulceration, and erosion. Crucially, it identified the intraluminal concretion as a phytobezoar composed of vegetable fibers with dystrophic calcification. Available hematoxylin and eosin (H&E)-stained microphotographs separately document severe ulcerative-necrotizing inflammation, a follicular inflammatory background, recognizable vegetable structures, and fields designated by the pathologist as showing associated dystrophic calcification (Figure 5). The report did not specify microscopic perforation; therefore, perforation is not asserted.

**Figure 5:**
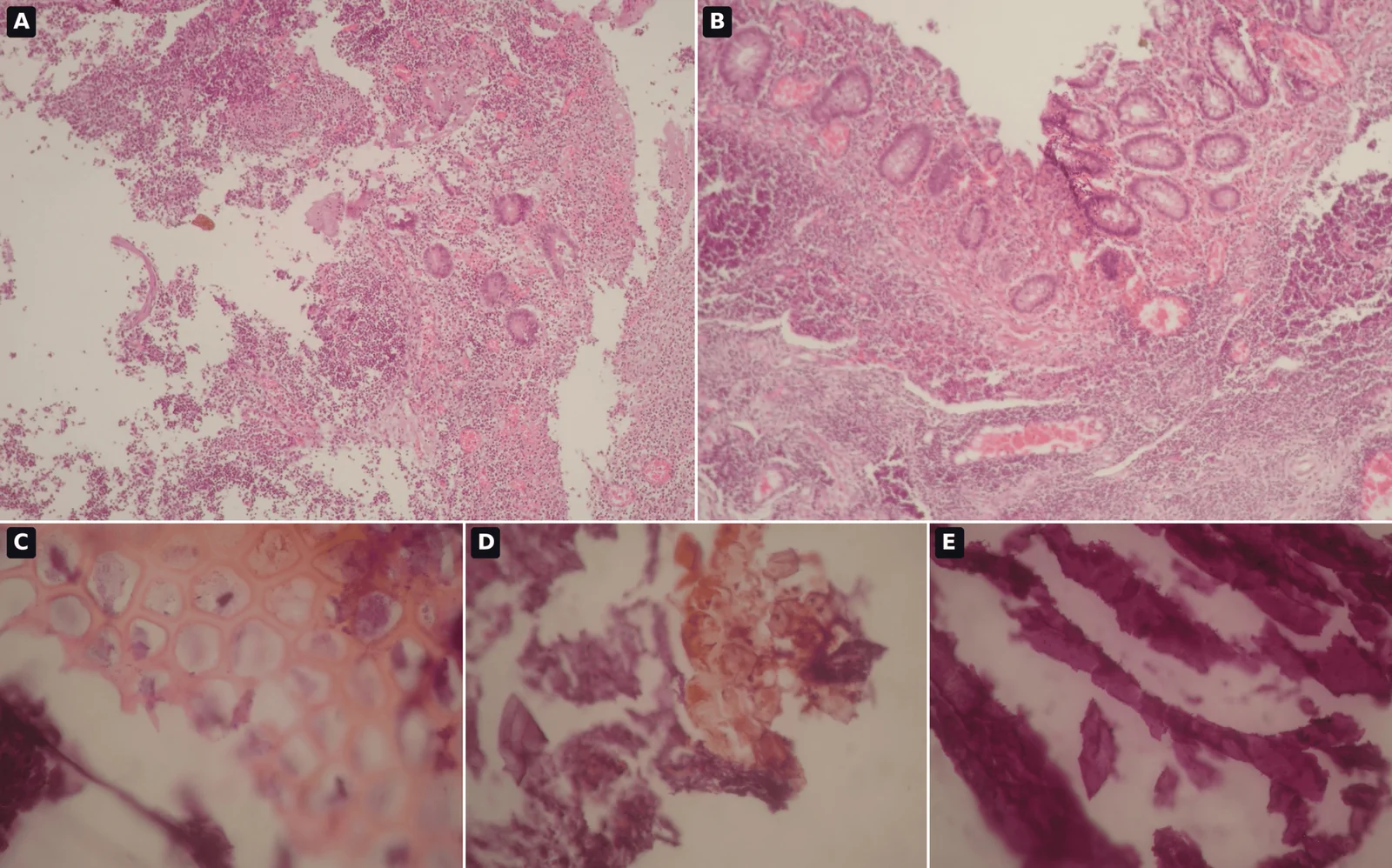
Histopathologic correlation of the inflamed appendix and the calcified phytobezoar. (A) Appendiceal mucosa with severe acute ulcerative-necrotizing and suppurative inflammation. (B) Separate section showing the follicular inflammatory background described by the pathologist. (C) Recognizable polygonal plant-derived structures with defined cell walls. (D) Vegetable tissue admixed with material reported by the pathologist as dystrophic calcification. (E) Additional field designated by the pathologist as illustrating dystrophic calcification. Hematoxylin and eosin (H&E) staining; reported magnification, 20× for all panels. These images support the reported pathologic diagnosis.

Postoperative course and follow-up

The patient received ceftriaxone and metronidazole. During the first postoperative 24 hours, the Blake drain yielded approximately 50 mL of purulent fluid. Subsequent clinical evolution was favorable, with no reported fever or leukocytosis, and she was discharged with the drain in place; the exact discharge date and antibiotic duration were not provided. At the postoperative day-seven outpatient visit, she remained clinically well, and the drain had no output, permitting removal. Reassessment on postoperative day 12 again documented satisfactory recovery without reported postoperative problems. The relative clinical sequence is summarized in Table 2.

**Table 2:** Clinical timeline. Clinical timeline from admission through urgent appendectomy, postoperative Blake drainage, drain removal on day 7, and follow-up on day 12.

Period	Verified clinical course
Presentation (~1 day of symptoms)	Severe right lower quadrant pain, nausea, vomiting; localized peritoneal signs; neutrophilic leukocytosis; radiopaque appendiceal-region focus and inflamed appendix on noncontrast CT.
Urgent laparoscopy	Severely inflamed enlarged appendix and localized basal periappendiceal purulent collection; aspirated after opening; appendectomy with ultrasound-based mesoappendix division, two endoloops, and Blake drain placement.
Postoperative day 1	Approximately 50 mL of purulent Blake drainage; ceftriaxone and metronidazole; clinically stable with subsequent favorable evolution.
Postoperative day 7	No drain output and satisfactory clinical recovery; Blake drain removed.
Postoperative day 12	Additional outpatient evaluation documented ongoing favorable postoperative recovery without reported problems.

## Discussion

The case is distinctive primarily because the presumed radiologic appendicolith had a tissue-confirmed vegetal composition, with dystrophic calcification, and was associated with severe appendiceal inflammation and a localized basal abscess. The term phytobezoar is based on the pathologist's explicit final diagnosis rather than the size or external appearance of the concretion. Size alone is not a defining criterion for a bezoar: small aggregates of indigestible plant material in the appendiceal lumen have already been described as microphytobezoars [2]. Accordingly, the 1.7 × 1.2 cm concretion in this patient is better described as a calcified appendiceal phytobezoar than as a “giant” appendicolith; the latter term is variably defined in published reports and adds little to the verified histologic finding.

Published examples establish precedent without diminishing the clinical value of the present documentation. In Engin et al.'s appendectomy series, seven cases contained undigested plant residue, but appendiceal inflammation was documented in only two [2]. A systematic review of foreign bodies in adults included 64 case reports and illustrated that ingested objects found in the appendix range from seeds to sharp or metallic materials, with varied clinical presentations [3]. Redden and Ghadiri described perforated appendicitis in an adolescent with a hair-containing appendiceal trichobezoar; that case involved a different bezoar substrate and cannot be considered an exact phytobezoar analogue [4]. The present case contributes a longitudinal, multimodal record of a comparatively large vegetal concretion whose calcified nature was visible before surgery and whose vegetal composition was confirmed after resection. A claim that this is the first such case would not be justified by the available evidence.

The distinction between a phytobezoar and a conventional appendicolith requires more than visual inspection of imaging. Appendicoliths are compositionally heterogeneous; prospective analysis using gross examination and advanced imaging has demonstrated variation in their structure and mineral composition [5]. Furthermore, a published pediatric case described a retained calcified appendicolith that, on pathologic evaluation, contained degenerated vegetable matter and food particles, illustrating that vegetal content can occur in a lesion still termed an appendicolith [6]. For that reason, isolated vegetable fragments do not automatically establish a phytobezoar. In the current specimen, the pathologist specifically classified the concretion as a phytobezoar formed by vegetable fibers and dystrophic calcification, while the gross photographs showed a discrete, organized intraluminal mass. The combination of that diagnostic wording and direct microphotography justifies the terminology used here without suggesting that radiology alone could distinguish this entity from a fecalith.

The patient also had complicated appendicitis despite a relatively short history of recognized symptoms. The documented presentation was approximately one day after symptom onset, yet surgery revealed a basal purulent collection and histology demonstrated severe suppurative-necrotizing inflammation. Symptom duration is not identical to the duration of luminal obstruction or microscopic inflammation. Thus, it is reasonable to discuss a possible contribution from prolonged, previously unrecognized impaction or a rapidly progressive inflammatory course, but not to conclude that the phytobezoar itself formed or that the entire pathologic process evolved within 24 hours. The observed ulcerated and fibrotic stenosis near the appendiceal orifice is compatible with a focal obstructive process, although the temporal relationship between stenosis, impaction, and inflammation cannot be reconstructed from a single specimen.

Existing studies strengthen the relevance of an obstructing appendiceal concretion while imposing important limits on inference. In a cohort of 3,085 patients with CT-diagnosed appendicitis, Sula et al. found complicated disease in 47.1% of patients with an appendicolith compared with 21.5% of those without one; larger diameter and basal location were associated with greater severity in the imaging reassessment subgroup [7]. A separate adult CT-pathology cohort also associated appendicoliths with complicated appendicitis, although diameter thresholds showed low specificity and should not be interpreted as deterministic [8]. More recent retrospective work again found an association between appendicoliths and complicated appendicitis but highlighted appendiceal diameter, fat stranding, and periappendiceal air as additional discriminating features [9]. These data were obtained predominantly in patients with radiologically defined appendicoliths, not histologically verified calcified phytobezoars. They therefore support the plausibility of obstruction-related severe disease but cannot quantify this patient's risk or prove causation by the vegetable material.

From an imaging perspective, the matching radiographic and tomographic densities are consistent with the dystrophic calcification identified in the phytobezoar. Plain films showed an unusually conspicuous focal opacity, and the noncontrast CT localized the concretion to the inflamed appendix. Nonetheless, the measurements varied among planes and modalities because the images sampled or projected different axes. The pathologic specimen measurement of 1.7 × 1.2 cm should serve as the definitive gross size reported for the lesion. Formal radiologic review was unavailable, so an additional retrospective diagnostic interpretation beyond the provided images is not implied.

The operative strategy addressed the proven localized intra-abdominal infection rather than simply the radiologic presence of a stone. Careful laparoscopic exposure demonstrated an intact base adjacent to the purulent collection; drainage, aspiration, source control, appendectomy, and two-loop stump closure were completed without observed macroscopic stump leakage. The Blake drain was a surgeon-selected response to the localized purulent process and should not be interpreted as evidence that routine drainage is necessary after all complicated appendectomies. The contemporary WSES recommendations favor laparoscopic surgery when appropriate and short postoperative antibiotic courses after adequate source control in complicated disease [1]. Because this patient's antibiotic dose and duration were not supplied, their consistency with a particular guideline duration cannot be assessed.

The case should also not be used to infer that a comparable uncomplicated appendicitis presentation necessarily requires surgery. In the Comparison of Outcomes of Antibiotic Drugs and Appendectomy (CODA) randomized trial, patients receiving antibiotics were more likely to undergo subsequent appendectomy when an appendicolith was present, and this subgroup accounted for the increased complication rate with antibiotic treatment [10]. However, CODA's findings about treatment choices are not directly interchangeable with those for an already recognized periappendiceal abscess and suppurative-necrotizing appendicitis. In this patient, the decision for operative management reflected localized peritoneal signs, a large obstructing concretion, and the clinical concern for complicated infection, later verified by operative findings.

The principal strength of this report is the complete sequential correlation among plain radiographs, noncontrast CT, laparoscopic findings, gross specimen photography, and histology showing vegetal structures and dystrophic calcification. Limitations include the absence of a formal radiologist's report in the provided record and uncertainty surrounding the actual onset of luminal obstruction. In addition, a single case cannot establish whether the phytobezoar's composition or diameter caused the observed abscess or accelerated the illness. Those limitations favor the more precise conclusion that the lesion was strongly associated anatomically and temporally with the documented complicated appendicitis.

## Conclusions

A calcified appendiceal phytobezoar can be radiographically indistinguishable from a conventional appendicolith yet have a substantially different histologic composition. In this patient, a 1.7 × 1.2 cm phytobezoar composed of vegetable fibers and dystrophic calcification was found within an enlarged, severely inflamed appendix in association with a localized purulent collection near the base. The diagnosis was established through gross and histopathologic assessment after laparoscopic appendectomy rather than on imaging alone.

This case demonstrates the clinical value of preserving the excised concretion and correlating radiographic, operative, and microscopic findings when an unusually conspicuous appendiceal calcification accompanies complicated appendicitis. Although the patient reported approximately one day of symptoms, the actual duration and sequence of the underlying obstructive and inflammatory processes remain uncertain.
